# HMG-CoA Reductase Inhibitors Attenuate Neuronal Damage by Suppressing Oxygen Glucose Deprivation-Induced Activated Microglial Cells

**DOI:** 10.1155/2019/7675496

**Published:** 2019-02-17

**Authors:** Dan Lu, Lingling Shen, Hongcheng Mai, Jiankun Zang, Yanfang Liu, Chi-kwan Tsang, Keshen Li, Anding Xu

**Affiliations:** ^1^The First Affiliated Hospital, Jinan University, Guangzhou, China; ^2^Department of Neurology and Stroke Center, The First Affiliated Hospital, Jinan University, Guangzhou, China; ^3^Clinical Neuroscience Institute of Jinan University, Guangzhou, China

## Abstract

Ischemic stroke is usually followed by inflammatory responses mediated by microglia. However, the effect of statins on directly preventing posthypoxia microglia inflammatory factors to prevent injury to surrounding healthy neurons is unclear. Atorvastatin and rosuvastatin, which have different physical properties regarding their lipid and water solubility, are the most common HMG-CoA reductase inhibitors (statins) and might directly block posthypoxia microglia inflammatory factors to prevent injury to surrounding neurons. Neuronal damage and microglial activation of the peri-infarct areas were investigated by Western blotting and immunofluorescence after 24 hours in a middle cerebral artery occlusion (MCAO) rat model. The decrease in neurons was in accordance with the increase in microglia, which could be reversed by both atorvastatin and rosuvastatin. The effects of statins on blocking secretions from posthypoxia microglia and reducing the secondary damage to surrounding normal neurons were studied in a coculture system *in vitro*. BV2 microglia were cultured under oxygen glucose deprivation (OGD) for 3 hours and then cocultured following reperfusion for 24 hours in the upper wells of transwell plates with primary neurons being cultured in the bottom wells. Inflammatory cytokines, including tumor necrosis factor-*α* (TNF-*α*), interleukin-1*β* (IL-1*β*), and cyclooxygenase-2 (COX2), which are activated by the nuclear factor-kappa B (NF-*κ*B) signaling pathway in OGD-induced BV2 microglia, promoted decreased release of the anti-inflammatory cytokine IL-10 and apoptosis of neurons in the coculture systems according to ELISA and Western blotting. However, pretreatment with atorvastatin or rosuvastatin significantly reduced neuronal death, synaptic injury, and amyloid-beta (A*β*) accumulation, which might lead to increased low-density lipoprotein receptors (LDLRs) in BV2 microglia. We concluded that the proinflammatory mediators released from postischemia damage could cause damage to surrounding normal neurons, while HMG-CoA reductase inhibitors prevented neuronal apoptosis and synaptic injury by inactivating microglia through blocking the NF-*κ*B signaling pathway.

## 1. Introduction

Ischemic stroke, accounting for eighty percent of all strokes, is one of the most common diseases and a leading cause of death and disability worldwide [1–3]. It is usually followed by inflammatory responses mediated by microglia [4, 5]. 3-Hydroxy-3-methylglutaryl coenzyme A (HMG-CoA) reductase inhibitors (statins) are traditional drugs widely used for secondary prevention of ischemic stroke by lowering lipids [6] and reducing inflammation [7, 8]. In addition, some studies have reported that pretreatment with HMG-CoA reductase inhibitors in animal stroke models [9] is effective for improving both short-term [10] and long-term functional outcomes in clinical trials [11]. However, how to inhibit the secondary and morphological injury of healthy neurons in the peri-infarct region must still be explored. While inflammation-induced hypoxia-ischemia neuronal damage has been previously reported [2, 12], the direct effect of secretions from microglia after cerebral ischemia on healthy neurons will be studied, which we believe may be a possible cause of penumbra formation. Actually, atorvastatin has no direct neuron protection effects [13], while rosuvastatin induces delayed preconditioning against oxygen-glucose deprivation in cultured cortical neurons [14]. That implies that the neuron loss inhibited by statins does not completely follow the same mechanism as that on neurons. According to the anti-inflammatory function of statins, we reason that the same mechanism of the common statins in neuroprotection under brain ischemia might be antineuroinflammation by inhibiting the secretion of microglia activation. This study will elucidate new targets for determining how the inhibition of microglial secretion by statin therapy can prevent deterioration of healthy neurons.

Microglia are the principal immune cells in the brain, representing a continuum of different phenotypes [15]. Therein, emerging studies have concentrated on the regulatory mechanisms that underlie the activation of microglia and have aimed at inhibiting the microglia activation to secrete proinflammatory cytokines such as tumor necrosis factor-*α* (TNF-*α*), interleukin-1*β* (IL-1*β*), and cyclooxygenase-2 (COX2) to inhibit secondary inflammation injury after ischemia in cells [16] and to facilitate protective microglia to secrete anti-inflammatory cytokines, such as IL-10, which my facilitate anti-inflammation, axonal outgrowth and promote neuron recovery [17, 18].

Statin therapy induces competitive inhibition of cholesterol synthesis by upregulating low-density lipoprotein receptors (LDLRs) and lowering low-density lipoprotein (LDL) in the peripheral blood system [19]. LDLR is a cell surface receptor of LDL and causes hypercholesteremia and risk of cardiovascular and cerebrovascular diseases, including cerebral stroke, by acting in the catabolism of LDL-C by endocytosis via clathrin-coated pits [19, 20]. Studies have shown that LDLR is also expressed in microglia and decreased expression of LDLR led to increased circulating LDL and neuroinflammation in Alzheimer's disease models [21–23]. Recent studies have also discovered that modulation of LDLR may be a safe and efficient therapy for AD by accelerating amyloid-beta (A*β*) clearance to inhibit neuronal apoptosis [24, 25]. Vascular and neurodegenerative pathologies contribute to A*β* accumulation and cause further neurotoxicity due to proinflammation, apoptosis, and synaptic injury pathways [26–29]. The immune system may contribute to the outcome of infarction [30]. As a fact, the observation study showed that a high percentage of patients suffered from ischemic stroke subsequently to develop AD, which suggests that there is a strong link between the pathologies of stroke and AD. In addition, both hypoxia and ischemic injury increase the production of A*β* by hypoxia-inducible factor 1*α* (HIF1*α*) activating the increase of BACE1 expression [31], which further confirms the link between AD and stroke [32]. There is also evidence that HMG-CoA reductase inhibitors exert anti-inflammatory effects induced by A*β* [33].

Although statin therapy can induce anti-inflammatory effects in many nervous system diseases, the direct effect of statins on preventing posthypoxia microglial inflammatory factors to minimize injury to surrounding healthy neurons remains unclear. Thus, we investigated whether two common statins, atorvastatin and rosuvastatin, prevented secretion by posthypoxia microglia to block secondary damage to surrounding normal neurons in vitro. In clinical administration, prior statin treatment is associated with lower stroke severity and better outcomes in acute ischemic stroke patients and statin pretreatment in patients with acute large artery atherosclerosis appears to be associated with better early outcomes regarding neurologic improvement, disability, survival, and stroke recurrence [34].

## 2. Materials and Methods

### 2.1. Middle Cerebral Artery Occlusion (MCAO) Model

A total of 45 male Sprague Dawley rats (200–250 g) were purchased from Guangdong Medical Laboratory Animal Center, Guangzhou, China. Thirty-five rats were subjected to MCAO, and 10 rats served as sham-operated controls. The MCAO model was developed as previously described [35]. Briefly, the rats were anesthetized with 10% chloral hydrate (BBI Life Sciences, Shanghai, China, 300 *μ*L/100 g) by intraperitoneal injection. The left middle cerebral artery was exposed through a subtemporal craniectomy and occluded by electrocoagulation under an operating microscope. Sham-operated animals underwent the same surgical procedures apart from electrocoagulation. Before operation, rats in the drug treatment and vehicle groups were intraperitoneally injected with rosuvastatin (Sigma, St. Louis, MO, USA, 5 mg/mL in normal saline, 5 mg/kg/d) or atorvastatin (Sigma, St. Louis, MO, USA, dissolved in DMSO and diluted in normal saline to 10 mg/mL, 10 mg/kg/d) and isomolar vehicle for three days. The animal experimental protocols were approved by the Animal Care and Use Committee of Jinan University. All experimental protocols involving rats were approved by the ethical committee of Jinan University and performed in accordance with approved guidelines and regulations.

### 2.2. 2,3,5-Triphenyltetrazolium Chloride (TTC) Staining

Five MCAO rats were deeply anesthetized with 10% chloral hydrate (600 *μ*L/100 g). The brains were quickly removed and chilled at -20°C for 20 min; the frozen brains were then coronally sliced into six sections (2 mm-thick sections). The sections were stained with 2% TTC (BBI Life Sciences, Shanghai, China) at 37°C for 30 min and fixed in 4% paraformaldehyde for 24 hours at 4°C. The stained slides were photographed using a camera (Leica, Heidelberger, Germany).

### 2.3. Cell Culture and Oxygen-Glucose Deprivation/Reperfusion (OGD) Model

Immortal BV2 murine microglial cells exhibit the phagocytosis and inflammatory activation characteristics of microglia [36]. For BV2 microglia culture, BV2 cells were incubated in the upper wells of 6-well transwell plates (40000/well) in Dulbecco's modified Eagle's medium (DMEM) (Gibco, New York, USA) with 10% foetal bovine serum (FBS) (Biological Industries, CT, USA) for 24 hours at 37°C in a 5% CO_2_ incubator.

For the OGD experiments, cells were incubated in Hank's buffer and placed in an oxygen-free culture induced by a hermetic bag and AnaeroPack-Anaero (Mitsubishi Gas Chemical, Tokyo, Japan) for 3 hours at 37°C. The cells were then reperfused and reincubated with DMEM with 10% FBS for 24 hours at 37°C in a 5% CO_2_ incubator.

For primary neuron culture, dissociated primary neurons of the cerebral cortex were collected from new-born mice within 12 hours of birth. Primary cortical neurons were incubated in the bottom wells of 6-well transwell plates (Corning, New York, USA) in DMEM/F12 (Gibco, New York, USA) for 4 hours at 37°C in a 5% CO_2_ incubator. The medium was then changed to neurobasal medium (Gibco, New York, USA) with 2% B27 (Gibco, New York, USA) and incubated for another 6 days at 37°C in a 5% CO_2_ incubator.

**Figure 1:**
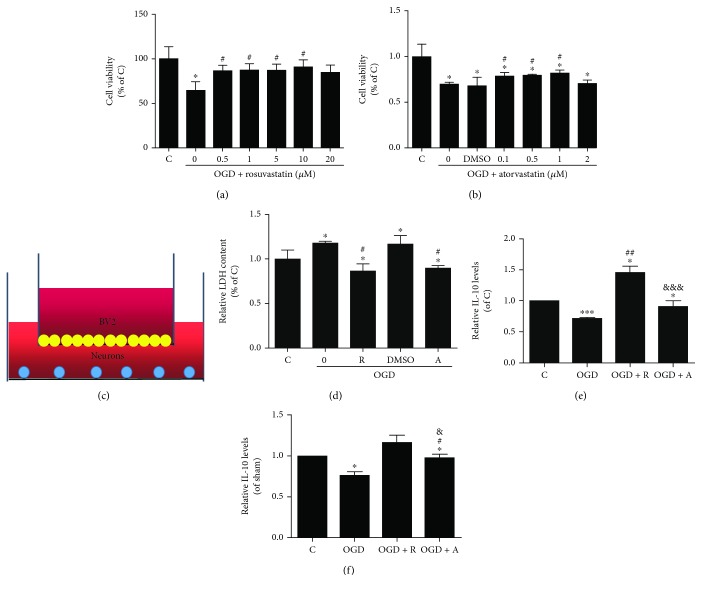
Rosuvastatin and atorvastatin suppressed the death of neurons by blocking inflammatory stimulation in the coculture systems. (a, b) Cell viability of BV2 cells for different concentrations of rosuvastatin and atorvastatin after OGD damage was measured by MTT assays. Values shown are expressed as the mean ± SEM as the ratio to the C group was assessed by one-way ANOVA followed by *L* test (homogeneity of variance was determined); *n* = 3. ^∗^*p* < 0.05 vs. the C group and ^#^*p* < 0.05 vs. the OGD group. (c) In the coculture systems, the BV2 cells in the upper wells were carefully placed on 6-well transwell plates and cocultured with neurons in the bottom wells. (d) Relative LDH levels in neurons cocultured with BV2 microglia were determined by LDH assays. (e, f) IL-10 expression in both BV2 cell culture supernatants and neuron culture supernatants was determined by ELISA. (b, c) Values shown are expressed as the mean ± SEM as the ratio to the C group was assessed by one-way ANOVA followed by LSD test (homogeneity of variance was determined), except that the expression of IL-10 of BV2 between-group differences was assessed by one-way ANOVA followed by Tamhane's *T*2 test (homogeneity of variance was not determined); *n* = 4. ^∗^*p* < 0.05 vs. the C group, ^∗∗^*p* < 0.01 vs. the C group, ^#^*p* < 0.05 vs. the OGD group, ^##^*p* < 0.05 vs. the OGD group, and ^&&&^*p* < 0.05 vs. the OGD group + R.

To study the effect of microglia secretion on neurons, coculture systems were set up as follows. After OGD for 3 hours and subsequent reperfusion for 24 hours at 37°C in a 5% CO_2_ incubator, hypoxic BV2 cells were carefully placed in the upper wells of 6-well transwell plates and cocultured with primary neurons in the bottom wells (Figure 1(c)).

### 2.4. Cell Viability Assays

The cell viability of BV2 cells was determined using methylthiazolyldiphenyl-tetrazolium (MTT) assays. A total of 5000 BV2 cells/well were incubated in 96-well plates in DMEM with 10% FBS for 12 hours at 37°C in a 5% CO_2_ incubator. Before inducing OGD, BV2 cells were pretreated with different doses of atorvastatin (0.1, 0.5, 1, and 2 *μ*M and the isomolar DMSO vehicle) or rosuvastatin (0, 0.5, 1, 5, 10, and 20 *μ*M) for another 24 hours. After OGD culture, the medium was discarded and the MTT reagent (20 *μ*L of 5 mg/mL solution, Sigma, USA) was added. The plate was incubated for 1 hour, and the MTT reagent of each well was changed with DMSO (150 *μ*L) to dissolve the insoluble purple substance. Next, the plate was incubated at room temperature for 10 min. The absorbance was measured at 570 nm using a microplate reader (Varioskan Flash, Thermo, Finland).

The cell survival of neurons was determined by a lactate dehydrogenase (LDH) assay after coculture of 10000 BV2 microglia and 10000 neurons in the six-well plates in neurobasal medium with 2% B27 for 5 days at 37°C in a 5% CO_2_ incubator [37]. Before coculture, the medium in the neuron culture was changed with fresh neurobasal medium with 2% B27. After OGD culture, the amount of LDH released from the neurons was measured by an LDH activity assay kit (Wanleibio, Liaoning, China) according to the manufacturer's protocol. The absorbance was measured at 440 nm using a microplate reader.

### 2.5. Enzyme-Linked Immunosorbent (ELISA) Assay

The relative levels of IL-10 in supernatants of the neuron cultures of the coculture systems were measured using a mouse IL-10 ELISA Kit (Elabscience, Hangzhou, China) according to the manufacturer's protocol. The mouse inflammation standard was used as the standard line. First, 100 *μ*L of each sample was added to each well for 40 min. After removing the liquid, 100 *μ*L of biotinylated detection antibody was added and the cultures were incubated for 1 hour at 37°C. Then, 100 *μ*L of HRP conjugate was added and the cultures were incubated for another 30 min at 37°C. Finally, 90 *μ*L of substrate reagent was added for 15 min, followed by 50 *μ*L of stop solution. The color in each microwell changed from blue to yellow. The OD value was immediately read at 450 nm. The concentrations of inflammatory factors were measured by the standard line. The relative levels of IL-10 were compared to those of the normal group.

### 2.6. Immunofluorescence Assays

Cell samples were fixed in 4% paraformaldehyde for 30 min at room temperature, while 10 *μ*m slides from the animals' frozen brains were fixed for 24 hours after heart perfusion. The immunofluorescence experiments were similar for the cells and brain slides. After incubation in 5% bovine serum albumin (BSA) and 0.2% Triton X-100 in phosphate-buffered saline (PBS) for 1 hour at room temperature, ionized calcium-binding adapter molecule 1 (Iba-1) (1 : 250; Santa Cruz Biotechnology, Texas, USA), neuron-specific nuclear protein (NeuN) (1 : 250; Abcam, MA, USA), LDLR (1 : 250; Abcam, MA, USA), microtube-associated protein (1 : 250; Abcam, MA, USA), and A*β* (1 : 250; Abcam, MA, USA) were applied overnight at 4°C and the corresponding secondary antibodies (1 : 250; Yeasen, Shanghai, China) were applied for 1 hour at room temperature. Slides were then counterstained with 4,6-diamidino-2-phenylindole dihydrochloride (DAPI) (Thermo Fisher, New York, USA) for 15 min at room temperature. Immunofluorescence images were captured under a confocal microscope (Leica, Heidelberger, Germany), and the cells were counted by using Image-Pro® Plus (Version 6.0 for Windows™, National Institute of Health, Bethesda, MD, USA) [38].

### 2.7. Western Blotting Analysis

**Figure 2:**
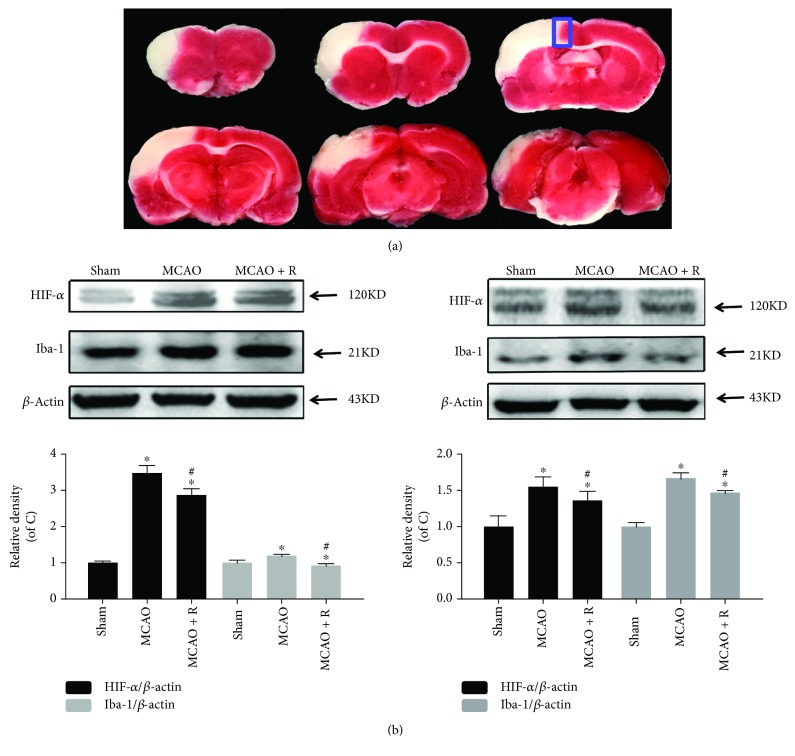
Microglial activation in cerebral ischemic rats. (a) An infarcted area appeared in the dorsolateral cortex at ischemic day 1 following occlusion of the distal striatal branch of the MCA with TTC staining. White areas indicate infarcted tissues, whereas red areas indicate normal tissues. The periphery of the ischemic area marked by the line was defined as the peri-infarct area. (b) HIF1-*α* and Iba-1 were determined in the ischemic cerebral cortex by Western blotting using respective antibodies. Quantified HIF1-*α* and Iba-1 are expressed as ratios to the sham group. Values shown are expressed as the mean ± SEM, which were assessed by one-way ANOVA followed by LSD test (homogeneity of variance was determined); *n* = 5. ^∗^*p* < 0.05 vs. the sham group and ^#^*p* < 0.05 vs. the MCAO group.

The expression of proteins of BV2 cells and neurons cultured in the coculture systems or brain tissues (the peri-infarct areas shown in Figure 2(a)) was analyzed by Western blotting. First, protein concentration was determined by using a BCA protein assay kit (Beyotime, Shanghai, China). The protein in each sample was separated by SDS/PAGE (Beyotime, Shanghai, China) and transferred to nitrocellulose membranes (NC, GE Healthcare Life Sciences, PA, USA). The membrane was blocked in 5% nonfat milk for 1 hour at room temperature. Then, NC membranes were incubated with hypoxia-inducible factor-1*α* (HIF1-*α*) (1 : 1000; Cell Signaling Technology, MA, USA), Iba-1 (1 : 1000; Santa Cruz Biotechnology, Shanghai, China), altered response to gravity1 (Arg1) (1 : 1000; Cell Signaling Technology, MA, USA), TNF-*α* (1 : 1000; Wanleibio, Shenyang, China), IL-1*β* (1 : 1000; Wanleibio, Shenyang, China), COX2 (1 : 1000; Cell Signaling Technology, MA, USA), pNF-*κ*B (1 : 1000; Cell Signaling Technology, MA, USA), pI*κ*Ba (1 : 1000; Cell Signaling Technology, MA, USA), postsynaptic density 95 (PSD95) (1 : 1000; Cell Signaling Technology, MA, USA), growth-associated protein 43 (GAP43) (1 : 1000; Wanleibio, Shenyang, China), cleaved-caspase 3 (cC3, 1 : 1000; Cell Signaling Technology, MA, USA), and *β*-actin (1 : 1000; Cell Signaling Technology, MA, USA) at 4°C for 16 hours. The membranes were then incubated for 1 hour with the corresponding horseradish peroxidase-conjugated IgG secondary antibody (Yeasen, Shanghai, China) at room temperature. Protein bands were visualized by using a chemiluminescence detection kit (Wanleibio, Liaoning, China).

### 2.8. Statistical Analysis

Data in the groups were analyzed with GraphPad Prism 5.0 (GraphPad Software, La Jolla, CA 92, USA) and one-way ANOVA in SPSS 19.0 (Abacus Concepts Inc., Chicago, IL, USA). All quantitative values were expressed as the mean ± standard error of the mean (SEM). Fisher's least significant difference (LSD) test was used for comparison between groups when homogeneity of variance was determined, while Tamhane's *T*2 test was used for comparison between groups when homogeneity of variance was not determined. Spearman test was used for correlation analysis of NeuN- and Iba-1-positive cells. Differences among groups were deemed significant at *p* < 0.05.

## 3. Results

### 3.1. Rosuvastatin and Atorvastatin Reduced the Number of Microglia and the Decrease in Neurons in MCAO Rats

Microglia are the principal immune cells of the brain [39] and are usually marked by Iba-1, an inflammation-associated Ca^2+^-binding protein produced from microglia [40]. The peri-infarct areas (Figure 2(a), the area of infarction is shown in white in the left cerebral cortex with TTC staining and the periphery of the ischemic area marked by the blue line was defined as the peri-infarct area) were collected for immunofluorescence and Western blotting. To investigate the neuroprotective effect of HMG-CoA reductase inhibitors in cerebral ischemia, HIF1-*α* was measured to determine whether HIF1-*α* was downregulated in brain cells in low-oxygen levels in the MCAO group pretreated with the HMG-CoA reductase inhibitor. In addition, the protein expression of Iba-1 assessed by Western blotting was promoted in the MCAO group, which was reduced in the MCAO surgery rats pretreated with rosuvastatin and atorvastatin (Figure 2(b), *p* < 0.05). This suggested that hypoxia is the potential stress for Iba-1 increasing.

**Figure 3:**
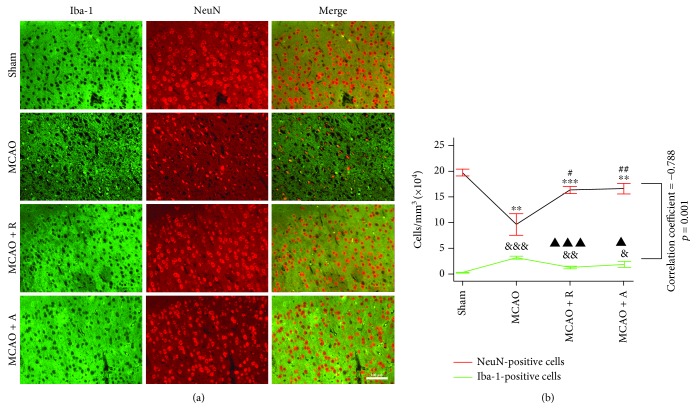
Rosuvastatin and atorvastatin decreased the number of microglia and reduced the decrease in neurons in MCAO rats. Ischemic injury resulted in increased numbers of Iba-1-positive cells and decreased numbers of neurons in the peri-infarct area cortex by immunofluorescence assays. Numbers of Iba-1 positive cells (cells/mm^3^) are expressed as compared to the sham group (^&^*p* < 0.05, ^&&^*p* < 0.01, and ^&&&^*p* < 0.001) and to the MCAO group (^#^*p* < 0.05, ^##^*p* < 0.01), while numbers of NeuN-positive cells are expressed as compared to the sham group (^∗∗^*p* < 0.01, ^∗∗∗^*p* < 0.001) and to the MCAO group (^▲^*p* < 0.05, ^▲▲^*p* < 0.01). These values were assessed by one-way ANOVA followed by LSD test (homogeneity of variance was determined); *n* = 5.

NeuN is a specific nuclear protein marker of neurons. The NeuN-positive cells in the peripheral area also showed similar patterns. The NeuN-positive cells significantly reduced in the MCAO group ((9.64 ± 1.04) × 10^4^ cells/mm^3^) as compared to the sham group ((19.74 ± 0.33) × 10^4^ cells/mm^3^) (^∗∗^*p* < 0.01), and three days of HMG-CoA pretreatment reversed the death of neurons induced by ischemic damage ((16.36 ± 0.33) × 10^4^ cells/mm^3^ in MCAO + R; (16.6 ± 0.52) × 10^4^ cells/mm^3^ in MCAO + A, Figure 3, ^#^*p* < 0.05, ^##^*p* < 0.01). Meanwhile, the number of Iba-1-positive cells was significantly increased, which might impose further inflammatory injury ((0.28 ± 0.04) × 10^4^ cells/mm^3^, ^&&&^*p* < 0.05) of the MCAO group compared with the sham group ((3.10 ± 0.20) × 10^4^ cells/mm^3^, Figure 1(b)) and three days of HMG-CoA pretreatment reversed the death of neurons induced by ischemic damage ((1.30 ± 0.12) × 10^4^ cells/mm^3^ in MCAO + R; (1.88 ± 0.29) × 10^4^ cells/mm^3^ in MCAO + A, Figure 3, ^▲^*p* < 0.05, ^▲▲▲^*p* < 0.05). By Spearman correlation analysis, there was a negative correlation between the two positive cell numbers (correlation coefficient = −0.788, *p* < 0.001). Therefore, rosuvastatin and atorvastatin attenuated neuronal death related to the hypoxia-induced microglia activation in MCAO rats.

### 3.2. Rosuvastatin and Atorvastatin Suppressed the Death of Neurons Caused by OGD-Induced BV2 Microglial Damage

To further identify the mechanism of the neuroprotective effect of the HMG-CoA reductase inhibitor, an *in vitro* experiment was designed. First, it was found that rosuvastatin and atorvastatin exerted protection in OGD-damaged BV2 cells compared with control cells and the most appropriate concentrations were 5 *μ*M rosuvastatin, which significantly increased cell viability in the ischemic condition (87.04 ± 6.98% vs. 64.86 ± 9.57% in the OGD group), and 1 *μ*M atorvastatin, which significantly increased cell viability in the ischemic condition (78.10 ± 4.40% vs. 67.52 ± 9.64% in the OGD group) *in vitro* (Figures 1(a) and 1(b), *p* < 0.05). It was observed that the hypoxic BV2 cells induced by OGD were neurotoxic to the primary neurons in the coculture systems (Figures 1(c) and 1(d), *p* < 0.05), which revealed that microglia had the ability to release some micromolecules that passed through the membrane of the transwell plate and contributed to neuronal injury after hypoxic stimulation. However, rosuvastatin and atorvastatin pretreatment reversed the BV2-induced neurotoxicity and protected neurons by blocking this pathway. As brain recovery and brain damage depend on the balance of proinflammation and anti-inflammation after ischemic stroke, it is necessary to determine the inflammatory cytokines involved in statin therapy. To explore whether HMG-CoA reductase inhibitors prevented neuronal damage by suppressing microglial activation and inflammatory stimulation, inflammatory factors were measured by ELISA and Western blotting. We next measured IL-10 and found that the level of IL-10 in the supernatants of neurons under the OGD condition was lower than that in the control group, while pretreatment with rosuvastatin and atorvastatin significantly upregulated IL-10 levels (Figures 1(f) and 1(g), *p* < 0.05).

### 3.3. Rosuvastatin and Atorvastatin Prevented Neuronal Damage by Blocking Inflammatory Stimulation in Coculture Systems

**Figure 4:**
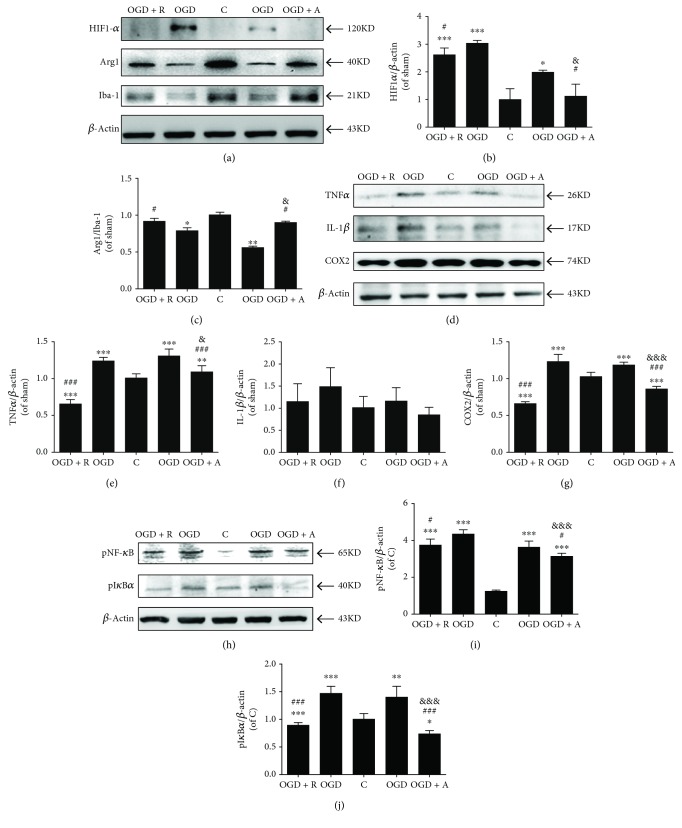
Rosuvastatin and atorvastatin prevented inflammatory activity in BV2 microglia. (a) HIF1-*α*, Arg1, and Iba-1 were determined in BV2 cells by Western blotting using the respective antibodies. Quantified (b) HIF1-*α*/*β*-actin and (c) Arg1/Iba-1 are expressed as ratios to the C group. (d, h) TNF-*α*, IL-1*β*, COX2, pNF-*κ*B, and pI*κ*B*α* were determined in BV2 cells by Western blotting using the respective antibodies. Quantified (e) TNF-*α*, (f) IL-1*β*, (g) COX2, (i) pNF-*κ*B, and (k) pI*κ*B*α* are expressed as ratios to the C group. Values shown are expressed as the mean ± SEM, and between-group differences were assessed by one-way ANOVA followed by LSD test (homogeneity of variance was determined), except that the expression of IL-1*β* between-group differences was assessed by one-way ANOVA followed by Tamhane's *T*2 test (homogeneity of variance was not determined); *n* = 3. ^∗∗∗^*p* < 0.001 vs. the C group, ^∗∗^*p* < 0.01 vs. the C group, ^∗^*p* < 0.05 vs. the C group, ^###^*p* < 0.001 vs. the OGD group, ^#^*p* < 0.05 vs. the OGD group, ^&&&^*p* < 0.001 vs. the OGD + R group, and ^&^*p* < 0.05 vs. the OGD + R group.

**Figure 5:**
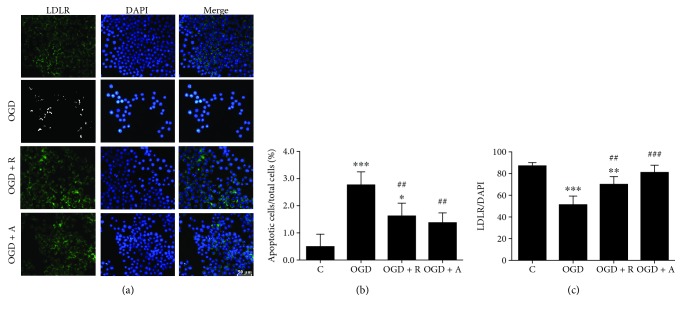
Rosuvastatin and atorvastatin increased LDLR expression in hypoxic BV2 cells. (a) OGD damage decreased the level of LDLR immunoreactivity in BV2 microglia. (b) Numbers of DAPI-positive cells are expressed as ratios to the C group (%). (c) Numbers of LDLR/DAPI-positive cells are expressed as ratios to the C group. Values shown are expressed as the mean ± SEM, and between-group differences were assessed by one-way ANOVA followed by LSD test (homogeneity of variance was determined); *n* = 3. ^∗∗∗^*p* < 0.001 vs. the C group, ^∗∗^*p* < 0.01 vs. the C group, ^∗^*p* < 0.05 vs. the C group, ^###^*p* < 0.001 vs. the OGD group, and ^##^*p* < 0.01 vs. the OGD group.

Studies showed that hypoxia can enhance the expression of HIF-1*α* in microglia and cause microglial activation on inflammation [41, 42]. During hypoxic HIF-1*α* binding to the hypoxic responsive element (HRE), the expression of NF-*κ*B in microglia is induced [43], to exaggerate inflammatory response by microglia and provoke excessive secretion of proinflammatory cytokines including IL-1*β* and TNF-*α* [44]. As shown in Figures 4(a) and 4(b), the phenotype of BV2 cells was assessed by Western blotting. Herein, it was the expression of the marker of protective microglia Arg1 [45], companied with the increase of the HIF-1*α* level of OGD-treated BV2 cells, that significantly decreased (^∗∗∗^*p* < 0.001 vs. C, ^#^*p* < 0.05 vs. OGD, and ^&^*p* < 0.05 vs. OGD + R) while the Arg1/Iba1 level was significantly increased by the rosuvastatin and atorvastatin treatment (^∗^*p* < 0.05 vs. C, ^∗∗^*p* < 0.01 vs. C, ^#^*p* < 0.05 vs. OGD, and ^&^*p* < 0.05 vs. OGD + R, Figure 4(c)). Furthermore, we sought to elucidate the protective mechanism of the HMG-CoA reductase inhibitors and analyze the levels of pNF-*κ*B, pI*κ*Ba, and their downstream responsive inflammatory factors, such as TNF-*α*, IL-1*β*, and COX2. The NF-*κ*B signaling pathway regulates both inflammation and apoptosis in cerebral ischemia. As expected, OGD-induced BV2 microglia exhibited significantly increased expression of inflammatory cytokines, such as TNF-*α* and COX2 (Figures 4(d)–4(g), ^∗∗∗^*p* < 0.001 vs. C, ^∗∗^*p* < 0.01 vs. C, ^###^*p* < 0.001 vs. OGD, ^&^*p* < 0.05 vs. OGD + R, and ^&&&^*p* < 0.001 vs. OGD + R; IL-1*β* exhibited a similar, nonsignificant trend), as well as enhanced activation of pI*κ*B*α* and pNF-*κ*B, which could be significantly decreased by rosuvastatin and atorvastatin treatment (Figures 5(h)–5(j), ^∗∗∗^*p* < 0.001 vs. C, ^∗∗^*p* < 0.01 vs. C, ^###^*p* < 0.001 vs. OGD, ^#^*p* < 0.05 vs. OGD, and ^&&&^*p* < 0.001 vs. OGD + R). Therefore, HMG-CoA reductase inhibitors prevent microglial activation-induced neuronal damage by blocking the pI*κ*B*α*/pNF-*κ*B signaling pathway and decreasing downstream inflammatory cytokines such as TNF-*α*, IL-1*β*, and COX2. Rosuvastatin, compared with atorvastatin, significantly reduced NF-*κ*B and IkBa phosphorylation for TNF-*α* reduction and Arg1 promotion under OGD condition.

### 3.4. Rosuvastatin and Atorvastatin Increased LDLR Expression in Hypoxic BV2 Cells

LDLR is expressed in the brain, especially in microglia. The levels of LDLR downregulation will lead to the increase of circulating LDL and neuroinflammation in Alzheimer's disease models, while as the potential targets of statins in ischemic stroke, the decrease of LDLR could be inhibited by statin treatment [24]. Herein, Western blot will be used to identify whether LDLR involved in the pathway of microglia activity induced neuron damage. As expected, the LDLR level was significantly decreased in OGD-induced BV2 cells compared with control cells (^∗∗∗^*p* < 0.01, ^∗∗^*p* < 0.01, and ^∗^*p* < 0.05), while rosuvastatin and atorvastatin upregulated the expression of LDLRs (^###^*p* < 0.001, ^##^*p* < 0.01), which may activate subsequent anti-inflammatory and neuroprotective effects (Figures 5(a) and 5(b), *p* < 0.05). Meanwhile, DAPI staining of cells was intact in the control group; OGD and reperfusion (OGD) caused aggravation of apoptosis and karyopyknosis, forming a number of particle spots in nuclei, while rosuvastatin and atorvastatin reduced particle spots in the OGD + R and OGD + A groups [46].

### 3.5. Rosuvastatin and Atorvastatin Exert Neuroprotective Effects against OGD Damage by Decreasing A*β* Accumulation in Neurons

**Figure 6:**
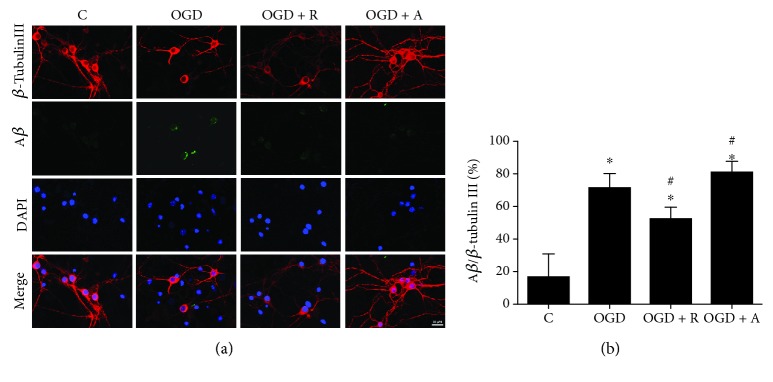
Rosuvastatin and atorvastatin exert neuroprotective effects against OGD damage by decreasing A*β* accumulation in neurons. (a) OGD damage resulted in increased levels of A*β* immunoreactivity in primary neurons. (b) Numbers of A*β*/*β*-tubulin III-positive cells are expressed as ratios to the C group. Values shown are expressed as the mean ± SEM, and between-group differences were assessed by one-way ANOVA followed by LSD test (homogeneity of variance was determined); *n* = 3. ^#^*p* < 0.05 vs. the OGD group and ^∗^*p* < 0.05 vs. the C group.

LDLRs contribute in A*β* clearance and inhibit neuronal apoptosis in vascular and neurodegenerative diseases, further leading to neurotoxicity by proinflammation, apoptosis, and synaptic injury pathways [29]. It is also known that proinflammatory cytokines secreted by microglia promote cognitive deficits in aged people and Alzheimer's disease (AD) patients [44, 47]. In addition, OGD-induced A*β* via the activation of the NF-*κ*B pathway could be detected by immunolabeling [38]. To investigate the role of A*β* in mediating the neuroprotective effect of HMG-CoA reductase inhibitors against OGD damage, we performed a study in primary neurons *in vitro*, and for detecting the A*β* accumulation, fluorescence immunolabeling for A*β* signaling cells was available [38, 48]. We found that the increased A*β* levels in the cytoplasm of neurons induced by OGD were significantly decreased by both rosuvastatin and atorvastatin treatment (Figures 6(a) and 6(b), ^∗^*p* < 0.05 vs. C, ^#^*p* < 0.05 vs. OGD).

### 3.6. Rosuvastatin and Atorvastatin Protected Neurons in the OGD Coculture System

**Figure 7:**
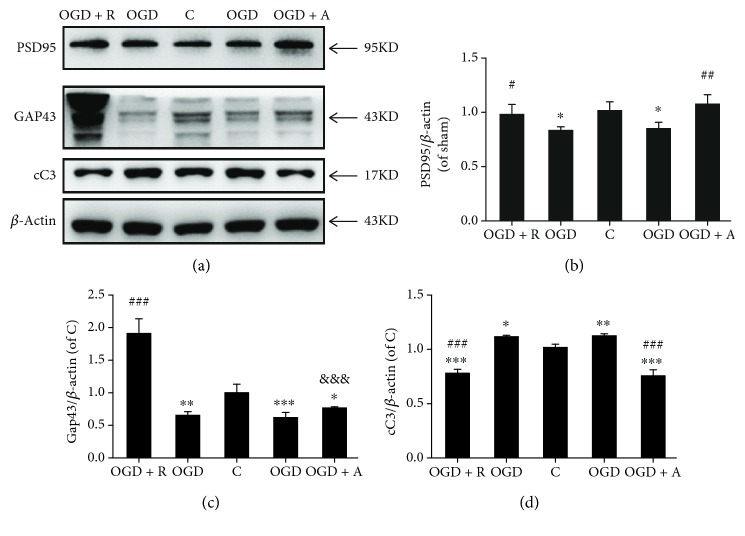
Rosuvastatin and atorvastatin protected neurons in the OGD coculture system: (a) PSD95, GAP43, and cC3 were determined in neurons by Western blotting using the respective antibodies. Quantified (b) PSD95, (c) GAP43, and (d) cC3 protein levels are expressed as ratios to the C group. Values shown are expressed as the mean ± SEM; *n* = 3. ^##^*p* < 0.01 vs. the OGD group, ^#^*p* < 0.05 vs. the OGD group, and ^∗^*p* < 0.05 vs. the C group.

As A*β* accumulation exerts neurotoxicity by apoptosis and synaptic injury pathways [29], we sought to determine whether antiapoptosis and antisynaptic injury are involved in the neuroprotective pathway of HMG-CoA reductase inhibitors. PSD95 and GAP43 were considered neuronal plasticity markers, as they play crucial roles in stimulating synapse formation and reconstruction [49]. Thus, PSD95 and GAP43 were identified by Western blotting. As expected, PSD95 and GAP43 were significantly decreased in OGD cells compared with control cells, while rosuvastatin and atorvastatin significantly increased the expression of these synaptic proteins (Figures 7(a)–7(c), *p* < 0.05). CC3 is known as a key marker of apoptosis status [50]. Rosuvastatin and atorvastatin also exhibited antiapoptotic effects by decreasing the upregulation of CC3 levels induced by OGD damage (Figures 7(a) and 7(d), *p* < 0.05). Therefore, the HMG-CoA reductase inhibitors suppressed the OGD-induced LDLR downregulation and A*β* accumulation and further protected neurons from apoptosis and synaptic injury.

## 4. Discussion

The available evidence has indicated that microglial activation results in the overproduction of proinflammatory cytokines, such as tumor necrosis factor-*α* (TNF-*α*), which may contribute to the development and progression of postischemic neuronal damage [51, 52]. Recent randomized clinical trials have demonstrated that HMG-CoA reductase inhibitors improve short-term [10] and long-term functional outcomes of ischemic stroke patients [11, 53], although statins have been proven to be neuroprotective in cerebral ischemia *in vivo* [8, 54, 55] and *in vitro* [56, 57]. A recent study showed that the administration of HMG-CoA reductase inhibitors was beneficial for neurodegenerative disorders through cholesterol-independent and cholesterol-dependent pathways and the effects were summarized as immunomodulation by suppression of inflammation and A*β* [2, 58, 59]. Atorvastatin and rosuvastatin, which are water- and lipid-soluble statins, respectively, are the traditional and most widely used drugs for the secondary prevention of ischemic stroke. In a previous study, atorvastatin did not protect from OGD and even exaggerated apoptotic cell death induced by several toxins [13], which differs from the studies *in vivo*; herein, in coculture studies of microglia and neuron under OGD condition, atorvastatin might improve the animal neuron survival by the mechanisms of antilipid, anti-inflammation, and so on. However, atorvastatin might have no direct neuron protection effects. Different from atorvastatin pretreatment, rosuvastatin induces delayed preconditioning against oxygen-glucose deprivation in cultured cortical neurons by decreasing ROS production and ATP levels [14]. However, whether the secretion itself from microglia affects the neurons and the associated effect of statin are unclear. This study showed the indirect protection on neurons by reducing microglia inflammation. Herein, we investigated the effects of these two physical classes of statins and the underlying neuroprotective mechanism by which they directly inhibit microglial secretion under hypoxia *in vitro*.

In the ischemic brain *in vivo*, activated microglia initially restrict the ischemic region and increase brain damage in the very beginning; however, proinflammatory mediators produced from microglia will activate more microglia to bring many secondary complications that prolong neuron damage in an acute phase of brain ischemia [60]. We observed the peri-infarct area of cerebral ischemia rats, where microglia were marked with Iba-1, and there were decreased neurons marked with NeuN. Therefore, we suspected that microglia suffered hypoxic damage released some micromolecules that transferred to the surrounding zones to induce injury to healthy neurons. Furthermore, the expression of Iba-1 was inhibited by HMG-CoA reductase inhibitors, including rosuvastatin and atorvastatin.

To further study the mechanism of the antineuroinflammation effect, we used posthypoxia BV2 microglia cocultured with normal neurons and evaluated the status of the neurons in transwell plates. In order not to harm neurons in the first place, we chose the protective doses of statins to inhibit microglia activation *in vivo*—which doses always affect the microglia viability to the same level as other antineuroinflammation drugs, such as “Celastrol” [61, 62]. It is hard to distinguish whether the neuroprotective effect is inhibited by antineuroinflammation or killing cells. Virtually, our speculation is focused on the indirect neuroprotective effect by antineuroinflammation. In vitro, it is necessary and easy to control the viability, because we should choose the doses which do not harm cell viability and antineuroinflammation to further verify the speculation that the neuroprotective effect of statin pretreatment is due to the indirect function of neuroinflammation. The minimal concentration of rosuvastatin resulting in neural cell damage is >10 *μ*M, while 0.5–10 *μ*M rosuvastatin is effective to inhibit the activation of microglia. Similarly, the minimal concentration of atorvastatin resulting in neurite loss is more than 2 *μ*M [63], while 0.1–1 *μ*M atorvastatin is effective to inhibit the activation of microglia. Thus, the dose of 10 *μ*M rosuvastatin and 1 *μ*M atorvastatin can be optimized to a concentration that does not cause neuronal injury.

Under OGD conditions, microglia, as macrophages, differentiated into the proinflammatory microglia, rather than the anti-inflammatory ones, and secreted proinflammatory mediators and cytokines, including IL-1*β*, TNF-*α*, and COX2, while atorvastatin inhibited inflammation via the expression of TNF-*α* and IL-1*β* and activation of caspase-3 [8, 64, 65]. Herein, the statins caused reduced TNF-*α*, IL-1*β*, and COX2 levels in the supernatants of hypoxia BV2 microglia, while damage to neurons was decreased according to the detected levels of LDH and upregulated levels of anti-inflammatory cytokine IL-10. Ischemia is known to increase the deleterious effect of A*β* production for synaptic impairment by induction of IL-1*β* and TNF-*α* of microglia, contributing to early cognitive impairment in Alzheimer's disease [66, 67]. We also observed A*β* accumulation and subsequent apoptosis and synaptic injury in neurons. PSD95 and GAP43 are related to synaptic plasticity and the expression of microtubule-associated protein 2 (MAP2) [68], while A*β* accumulation induces decreases in PSD95 and GAP43 until synaptic toxicity of neurons occurs [69]. With the administration of HMG-CoA reductase inhibitors, the levels of PSD95 and GAP43 decreased, accompanied by reduced A*β* accumulation. In addition, we observed reduced expression of CC3 following pretreatment with rosuvastatin and atorvastatin, supporting the conclusion that statins protected against hypoxia injury and, specifically, that statins increased the neuronal cell survival rate after ischemia.

Many studies have suggested that NF-*κ*B activation is involved in ischemic brain damage [70, 71]. We and other investigators have demonstrated that the neuroprotective effects of several drugs are associated with inhibition of NF-*κ*B activation and microglial inflammation [72, 73]. The present study showed that statins could regulate NF-*κ*B activation and the inflammatory response of microglia to cause a proinflammation response in neurons. NF-*κ*B transcription factors are usually present in the cytosol in an inactive state complexed with inhibitory I*κ*B proteins; activation occurs via phosphorylation of I*κ*B*α* at Ser32 and Ser36 followed by proteasome-mediated degradation, which results in the release and nuclear translocation of active NF-*κ*B in activated microglia. However, rosuvastatin and atorvastatin might prohibit the activation of I*κ*B*α* to inhibit the ischemia-induced activation of NF-*κ*B, and thus, the expression of proinflammatory mediators and cytokines, including IL-1*β*, TNF-*α*, and COX2, was inhibited. Paralleled with the decrease in inflammation, the enhanced expression of LDLR in microglia was prevented by both atorvastatin and rosuvastatin. Statins are cholesterol-lowering drugs and react in a complex manner with LDLRs, and many studies have shown that LDLRs decrease circulating LDL levels and prevent neuroinflammation in Alzheimer's disease models [21, 23]. Therefore, LDLRs might be regulated in microglia activation by both atorvastatin and rosuvastatin in ischemic stroke [74].

There were some limitations of the current study. BV2 cells are the best useful model to study microglia but also have many limitations, including different phenotypic, functional, and transcriptomic differences compared to primary microglia and even more with *in vivo* microglia; so, further studies should be validated *in vivo* at the future. Microglial secretion that caused morphological damage to the surrounding normal neurons should be verified by animal experiments. Furthermore, all secreted factors, in addition to the IL-1*β*, TNF-*α*, and COX2 cytokines, such as exosomes and microvesicles should be detected by microdialysis technology and proteomics analyses *in vivo*, which calls for further studies.

## 5. Conclusion

**Figure 8:**
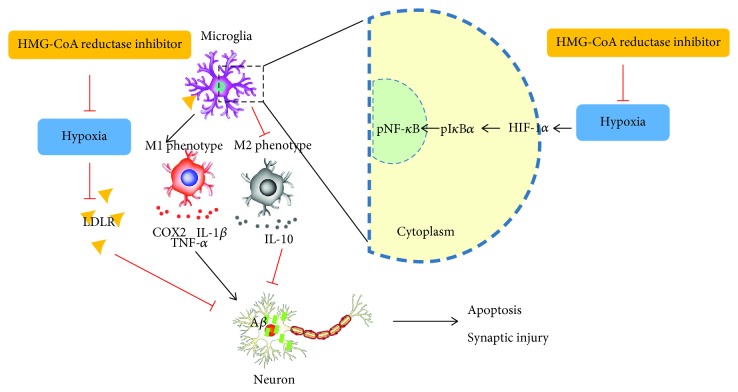
HMG-CoA reductase inhibitors prevent A*β* accumulation-induced apoptosis and synaptic injury in neurons by ameliorating the LDLR downregulation induced by OGD damage in microglia, suppressing microglia activity by blocking the pI*κ*B*α*/pNF-*κ*B signaling pathway and decreasing downstream inflammatory cytokines such as TNF-*α*, IL-1*β*, and COX2 and upregulating the anti-inflammatory cytokine IL-10.

In summary, our study concluded that the proinflammatory mediators released from postischemia damage could cause damage to normal neurons, while HMG-CoA reductase inhibitors prevented neuronal apoptosis and synaptic injury by inactivating microglia. The underlying mechanism is that HMG-CoA reductase inhibitors prevent A*β* accumulation-induced apoptosis and synaptic injury in neurons by ameliorating the LDLR downregulation induced by OGD damage in microglia, suppressing microglia activity by blocking the pI*κ*B*α*/pNF-*κ*B signaling pathway and decreasing downstream inflammatory cytokines such as TNF-*α*, IL-1*β*, and COX2 and upregulating the anti-inflammatory cytokine IL-10 (Figure 8).

## Data Availability

The data used to support the findings of this study are included within the article.

## References

[1] Jauch E. C., Saver J. L., Adams HP Jr (2013). Guidelines for the early management of patients with acute ischemic stroke: a guideline for healthcare professionals from the American Heart Association/American Stroke Association. *Stroke*.

[2] Moretti A., Ferrari F., Villa R. F. (2015). Neuroprotection for ischaemic stroke: current status and challenges. *Pharmacology & Therapeutics*.

[3] Wang Y., Liu M., Pu C. (2017). 2014 Chinese guidelines for secondary prevention of ischemic stroke and transient ischemic attack. *International Journal of Stroke*.

[4] Chen Y., Won S. J., Xu Y., Swanson R. A. (2014). Targeting microglial activation in stroke therapy: pharmacological tools and gender effects. *Current Medicinal Chemistry*.

[5] Stoll G., Jander S., Schroeter M. (1998). Inflammation and glial responses in ischemic brain lesions. *Progress in Neurobiology*.

[6] Hwong W. Y., Abdul Aziz Z., Sidek N. N. (2017). Prescription of secondary preventive drugs after ischemic stroke: results from the Malaysian National Stroke Registry. *BMC Neurology*.

[7] Griffin J. M., Kho D., Graham E. S., Nicholson L. F. B., O’Carroll S. J. (2016). Statins inhibit fibrillary *β*-amyloid induced inflammation in a model of the human blood brain barrier. *PLoS One*.

[8] Simani L., Naderi N., Khodagholi F., Mehrpour M., Nasoohi S. (2016). Association of long-term atorvastatin with escalated stroke-induced neuroinflammation in rats. *Journal of Molecular Neuroscience*.

[9] García-Bonilla L., Campos M., Giralt D. (2012). Evidence for the efficacy of statins in animal stroke models: a meta-analysis. *Journal of Neurochemistry*.

[10] Fang J. X., Wang E. Q., Wang W., Liu Y., Cheng G. (2017). The efficacy and safety of high-dose statins in acute phase of ischemic stroke and transient ischemic attack: a systematic review. *Internal and Emergency Medicine*.

[11] Kim J., Lee H. S., Nam C. M., Heo J. H. (2017). Effects of statin intensity and adherence on the long-term prognosis after acute ischemic stroke. *Stroke*.

[12] Kelly P., Prabhakaran S. (2017). Statins for neuroprotection after acute ischemic stroke: ASSORTed results but more trials needed. *Stroke*.

[13] Bösel J., Gandor F., Harms C. (2005). Neuroprotective effects of atorvastatin against glutamate-induced excitotoxicity in primary cortical neurones. *Journal of Neurochemistry*.

[14] Domoki F., Kis B., Gáspár T. (2009). Rosuvastatin induces delayed preconditioning against oxygen-glucose deprivation in cultured cortical neurons. *American Journal of Physiology-Cell Physiology*.

[15] Ransohoff R. M. (2016). Neuroinflammation: surprises from the sanitary engineers. *Nature*.

[16] Yang J., Zhao Y., Zhang L. (2018). RIPK3/MLKL-mediated neuronal necroptosis modulates the M1/M2 polarization of microglia/macrophages in the ischemic cortex. *Cereb Cortex*.

[17] Chen H., Lin W., Zhang Y. (2016). IL-10 promotes neurite outgrowth and synapse formation in cultured cortical neurons after the oxygen-glucose deprivation via JAK1/STAT3 pathway. *Scientific Reports*.

[18] Kanazawa M., Ninomiya I., Hatakeyama M., Takahashi T., Shimohata T. (2017). Microglia and monocytes/macrophages polarization reveal novel therapeutic mechanism against stroke. *International Journal of Molecular Sciences*.

[19] Polisecki E., Muallem H., Maeda N. (2008). Genetic variation at the LDL receptor and HMG-CoA reductase gene loci, lipid levels, statin response, and cardiovascular disease incidence in PROSPER. *Atherosclerosis*.

[20] Brown M. S., Goldstein J. L. (1986). A receptor-mediated pathway for cholesterol homeostasis. *Science*.

[21] Buga G. M., Frank J. S., Mottino G. A. (2006). D-4F decreases brain arteriole inflammation and improves cognitive performance in LDL receptor-null mice on a Western diet. *Journal of Lipid Research*.

[22] Katsouri L., Georgopoulos S. (2011). Lack of LDL receptor enhances amyloid deposition and decreases glial response in an Alzheimer's disease mouse model. *PLoS One*.

[23] Rutkowsky J. M., Lee L. L., Puchowicz M. (2018). Reduced cognitive function, increased blood-brain-barrier transport and inflammatory responses, and altered brain metabolites in LDLr −/−and C57BL/6 mice fed a Western diet. *PLoS One*.

[24] Thirumangalakudi L., Prakasam A., Zhang R. (2008). High cholesterol-induced neuroinflammation and amyloid precursor protein processing correlate with loss of working memory in mice. *Journal of Neurochemistry*.

[25] Yao L., Gu X., Song Q. (2016). Nanoformulated alpha-mangostin ameliorates Alzheimer's disease neuropathology by elevating LDLR expression and accelerating amyloid-beta clearance. *Journal of Controlled Release*.

[26] Desmond D. W., Moroney J. T., Sano M., Stern Y. (2002). Incidence of dementia after ischemic stroke: results of a longitudinal study. *Stroke*.

[27] Grau-Olivares M., Arboix A. (2009). Mild cognitive impairment in stroke patients with ischemic cerebral small-vessel disease: a forerunner of vascular dementia?. *Expert Review of Neurotherapeutics*.

[28] Schneider J. A., Bennett D. A. (2010). Where vascular meets neurodegenerative disease. *Stroke*.

[29] Popa-Wagner A., Buga A. M., Popescu B., Muresanu D. (2013). Vascular cognitive impairment, dementia, aging and energy demand. A vicious cycle. *Journal of Neural Transmission*.

[30] Iadecola C., Anrather J. (2011). The immunology of stroke: from mechanisms to translation. *Nature Medicine*.

[31] Guglielmotto M., Aragno M., Autelli R. (2009). The up-regulation of BACE1 mediated by hypoxia and ischemic injury: role of oxidative stress and HIF1*α*. *Journal of Neurochemistry*.

[32] Sun P., Esteban G., Inokuchi T. (2015). Protective effect of the multitarget compound DPH-4 on human SSAO/VAP-1-expressing hCMEC/D3 cells under oxygen-glucose deprivation conditions: an in vitro experimental model of cerebral ischaemia. *British Journal of Pharmacology*.

[33] Clarke R. M., O'Connell F., Lyons A., Lynch M. A. (2007). The HMG-CoA reductase inhibitor, atorvastatin, attenuates the effects of acute administration of amyloid-*β*_1–42_ in the rat hippocampus in vivo. *Neuropharmacology*.

[34] Tsivgoulis G., Katsanos A. H., Sharma V. K. (2016). Statin pretreatment is associated with better outcomes in large artery atherosclerotic stroke. *Neurology*.

[35] Zhu H., Zhang Y., Shi Z. (2016). The neuroprotection of liraglutide against ischaemia-induced apoptosis through the activation of the PI3K/AKT and MAPK pathways. *Scientific Reports*.

[36] Ries M., Loiola R., Shah U. N., Gentleman S. M., Solito E., Sastre M. (2016). The anti-inflammatory Annexin A1 induces the clearance and degradation of the amyloid-*β* peptide. *Journal of Neuroinflammation*.

[37] Li Z., Zeng Y., Chen X. (2016). Neural stem cells transplanted to the subretinal space of rd1 mice delay retinal degeneration by suppressing microglia activation. *Cytotherapy*.

[38] Zhang T., Wang H., Li Q., Huang J., Sun X. (2014). Modulating autophagy affects neuroamyloidogenesis in an *in vitro* ischemic stroke model. *Neuroscience*.

[39] McDonough A., Weinstein J. R. (2017). Correction to: neuroimmune response in ischemic preconditioning. *Neurotherapeutics*.

[40] Ito D., Imai Y., Ohsawa K., Nakajima K., Fukuuchi Y., Kohsaka S. (1998). Microglia-specific localisation of a novel calcium binding protein, Iba1. *Molecular Brain Research*.

[41] Huang T., Huang W., Zhang Z. (2014). Hypoxia-inducible factor-1*α* upregulation in microglia following hypoxia protects against ischemia-induced cerebral infarction. *NeuroReport*.

[42] Yang Z., Zhao T. Z., Zou Y. J., Zhang J. H., Feng H. (2014). Hypoxia Induces autophagic cell death through hypoxia-inducible factor 1*α* in microglia. *PLoS One*.

[43] Mukandala G., Tynan R., Lanigan S., O'Connor J. J. (2016). The effects of hypoxia and inflammation on synaptic signaling in the CNS. *Brain Sciences*.

[44] Wu Z., Zhu A., Takayama F. (2013). Brazilian green propolis suppresses the hypoxia-induced neuroinflammatory responses by inhibiting NF-*κ*B activation in microglia. *Oxidative Medicine and Cellular Longevity*.

[45] Xiang B., Xiao C., Shen T., Li X. (2018). Anti-inflammatory effects of anisalcohol on lipopolysaccharide-stimulated BV2 microglia via selective modulation of microglia polarization and down-regulation of NF-*κ*B p65 and JNK activation. *Molecular Immunology*.

[46] Sun Y., Zhu W., Zhou S., Wang Z., Chen X., Jia L. (2017). Exploring the model of PC12 apoptosis induced by OGSD/R through *in vitro* experiments. *Oncotarget*.

[47] Heneka M. T., O’Banion M. K., Terwel D., Kummer M. P. (2010). Neuroinflammatory processes in Alzheimer's disease. *Journal of Neural Transmission*.

[48] Clarke J. R., Lyra e Silva N. M., Figueiredo C. P. (2015). Alzheimer-associated A*β* oligomers impact the central nervous system to induce peripheral metabolic deregulation. *EMBO Molecular Medicine*.

[49] Ma J., Zhang Z., Su Y. (2013). Magnetic stimulation modulates structural synaptic plasticity and regulates BDNF–TrkB signal pathway in cultured hippocampal neurons. *Neurochemistry International*.

[50] Liu Y., Yang H., Jia G. (2018). The synergistic neuroprotective effects of combined rosuvastatin and resveratrol pretreatment against cerebral ischemia/reperfusion injury. *Journal of Stroke and Cerebrovascular Diseases*.

[51] Gao H. M., Hong J. S. (2008). Why neurodegenerative diseases are progressive: uncontrolled inflammation drives disease progression. *Trends in Immunology*.

[52] Zhang L., Dong L. Y., Li Y. J., Hong Z., Wei W. S. (2012). The microRNA miR-181c controls microglia-mediated neuronal apoptosis by suppressing tumor necrosis factor. *Journal of Neuroinflammation*.

[53] Yi X., Han Z., Wang C., Zhou Q., Lin J. (2017). Statin and aspirin pretreatment are associated with lower neurological deterioration and platelet activity in patients with acute ischemic stroke. *Journal of Stroke and Cerebrovascular Diseases*.

[54] Yang J., Pan Y., Li X., Wang X. (2015). Atorvastatin attenuates cognitive deficits through Akt1/caspase-3 signaling pathway in ischemic stroke. *Brain Research*.

[55] Rodríguez-Perea A. L., Gutierrez-Vargas J., Cardona-Gómez G. P., Guarin C. J. M., Rojas M., Hernández P. A. V. (2016). Atorvastatin modulates regulatory T cells and attenuates cerebral damage in a model of transient middle cerebral artery occlusion in rats. *Journal of Neuroimmune Pharmacology*.

[56] Lim J. H., Lee J. C., Lee Y. H. (2006). Simvastatin prevents oxygen and glucose deprivation/reoxygenation-induced death of cortical neurons by reducing the production and toxicity of 4-hydroxy-2E-nonenal. *Journal of Neurochemistry*.

[57] Sohn H. M., Hwang J. Y., Ryu J. H. (2017). Simvastatin protects ischemic spinal cord injury from cell death and cytotoxicity through decreasing oxidative stress: in vitro primary cultured rat spinal cord model under oxygen and glucose deprivation-reoxygenation conditions. *Journal of Orthopaedic Surgery and Research*.

[58] Hong K. S., Lee J. S. (2015). Statins in acute ischemic stroke: a systematic review. *Journal of Stroke*.

[59] Saeedi Saravi S. S., Saeedi Saravi S. S., Arefidoust A., Dehpour A. R. (2017). The beneficial effects of HMG-CoA reductase inhibitors in the processes of neurodegeneration. *Metabolic Brain Disease*.

[60] Gülke E., Gelderblom M., Magnus T. (2018). Danger signals in stroke and their role on microglia activation after ischemia. *Therapeutic Advances in Neurological Disorders*.

[61] Jiang M., Liu X., Zhang D. (2018). Celastrol treatment protects against acute ischemic stroke-induced brain injury by promoting an IL-33/ST2 axis-mediated microglia/macrophage M2 polarization. *Journal of Neuroinflammation*.

[62] Lu D., Liu Y., Mai H. (2018). Rosuvastatin reduces neuroinflammation in the hemorrhagic transformation after rt-PA treatment in a mouse model of experimental stroke. *Frontiers in Cellular Neuroscience*.

[63] Schulz J. G., Bosel J., Stoeckel M., Megow D., Dirnagl U., Endres M. (2004). HMG-CoA reductase inhibition causes neurite loss by interfering with geranylgeranylpyrophosphate synthesis. *Journal of Neurochemistry*.

[64] Li H. Y., Su Y. Y., Zhang Y. F., Liu Z. Q., Hua B. J. (2016). Involvement of peroxisome proliferator activated receptor-*γ* in the anti-inflammatory effects of atorvastatin in oxygen-glucose deprivation/reperfusion-stimulated RAW264.7 murine macrophages. *Molecular Medicine Reports*.

[65] Shao S., Xu M., Zhou J. (2016). Atorvastatin attenuates ischemia/reperfusion-induced hippocampal neurons injury via Akt-nNOS-JNK signaling pathway. *Cellular and Molecular Neurobiology*.

[66] Origlia N., Criscuolo C., Arancio O., Yan S. S., Domenici L. (2014). RAGE inhibition in microglia prevents ischemia-dependent synaptic dysfunction in an amyloid-enriched environment. *Journal of Neuroscience*.

[67] Origlia N., Bonadonna C., Rosellini A. (2010). Microglial receptor for advanced glycation end product-dependent signal pathway drives *β*-amyloid-induced synaptic depression and long-term depression impairment in entorhinal cortex. *Journal of Neuroscience*.

[68] Song J., Cheon S. Y., Lee W. T., Park K. A., Lee J. E. (2015). PKA inhibitor H89 (N-[2-p-bromocinnamylamino-ethyl]-5-isoquinolinesulfonamide) attenuates synaptic dysfunction and neuronal cell death following ischemic injury. *Neural Plasticity*.

[69] Manczak M., Kandimalla R., Fry D., Sesaki H., Reddy P. H. (2016). Protective effects of reduced dynamin-related protein 1 against amyloid beta-induced mitochondrial dysfunction and synaptic damage in Alzheimer's disease. *Human Molecular Genetics*.

[70] Stephenson D., Yin T., Smalstig E. B. (2000). Transcription factor nuclear factor-kappa B is activated in neurons after focal cerebral ischemia. *Journal of Cerebral Blood Flow & Metabolism*.

[71] Kunz A., Abe T., Hochrainer K. (2008). Nuclear factor-*κ*B activation and postischemic inflammation are suppressed in CD36-null mice after middle cerebral artery occlusion. *Journal of Neuroscience*.

[72] Paraiso H. C., Kuo P. C., Curfman E. T. (2018). Dimethyl fumarate attenuates reactive microglia and long-term memory deficits following systemic immune challenge. *Journal of Neuroinflammation*.

[73] Lu D., Zhu L. H., Shu X. M. (2015). Ginsenoside Rg1 relieves *tert*-Butyl hydroperoxide-induced cell impairment in mouse microglial BV2 cells. *J Asian Nat Prod Res*.

[74] Lin C. P., Huang P. H., Lai C. F., Chen J. W., Lin S. J., Chen J. S. (2015). Simvastatin attenuates oxidative stress, NF-*κ*B activation, and artery calcification in LDLR−/− mice fed with high fat diet via down-regulation of tumor necrosis factor-*α* and TNF receptor 1. *PLoS One*.

